# Scale effects and morphological diversification in hindlimb segment mass proportions in neognath birds

**DOI:** 10.1186/1742-9994-11-37

**Published:** 2014-05-06

**Authors:** Brandon M Kilbourne

**Affiliations:** 1 Institut für Spezielle Zoologie und Evolutionsbiologie, Friedrich-Schiller-Universität Jena, Erbertstraße 1, Jena 07743, Germany

## Abstract

**Introduction:**

In spite of considerable work on the linear proportions of limbs in amniotes, it remains unknown whether differences in scale effects between proximal and distal limb segments has the potential to influence locomotor costs in amniote lineages and how changes in the mass proportions of limbs have factored into amniote diversification. To broaden our understanding of how the mass proportions of limbs vary within amniote lineages, I collected data on hindlimb segment masses – thigh, shank, pes, tarsometatarsal segment, and digits – from 38 species of neognath birds, one of the most speciose amniote clades. I scaled each of these traits against measures of body size (body mass) and hindlimb size (hindlimb length) to test for departures from isometry. Additionally, I applied two parameters of trait evolution (Pagel’s λ and δ) to understand patterns of diversification in hindlimb segment mass in neognaths.

**Results:**

All segment masses are positively allometric with body mass. Segment masses are isometric with hindlimb length. When examining scale effects in the neognath subclade Land Birds, segment masses were again positively allometric with body mass; however, shank, pedal, and tarsometatarsal segment masses were also positively allometric with hindlimb length. Methods of branch length scaling to detect phylogenetic signal (i.e., Pagel’s λ) and increasing or decreasing rates of trait change over time (i.e., Pagel’s δ) suffer from wide confidence intervals, likely due to small sample size and deep divergence times.

**Conclusions:**

The scaling of segment masses appears to be more strongly related to the scaling of limb bone mass as opposed to length, and the scaling of hindlimb mass distribution is more a function of scale effects in limb posture than proximo-distal differences in the scaling of limb segment mass. Though negative allometry of segment masses appears to be precluded by the need for mechanically sound limbs, the positive allometry of segment masses relative to body mass may underlie scale effects in stride frequency and length between smaller and larger neognaths. While variation in linear proportions of limbs appear to be governed by developmental mechanisms, variation in mass proportions does not appear to be constrained so.

## Introduction

The relative proportions of limb segments are one of the most conspicuous aspects of whole limb morphology. In terms of segment lengths, the proportions of limbs have been extensively studied in major amniote groups, including mammals [1-5], non-avian dinosaurs [3,6-10], pterosaurs [11], birds [6,8,12-15], lizards [16-19], and turtles [20]. Within these groups, the relative lengths of limb segments have been linked to specializations for predominant habitat [12,15,20], biomechanical demands [3-5,7,21], and functional diversity [6,8].

Though bone masses have been studied in mammals [22] and birds [23], previous studies on masses of whole limb segments inclusive of both hard and soft tissues have focused primarily on ungulates and primates [24-33], otherwise receiving little attention. Thus, it remains unknown how size influences changes in the mass proportions of limbs within most amniote lineages. Yet the relative masses within limb segments are likely critical to terrestrial locomotion [34]. Less massive distal limb segments give the limb a more proximal concentration of mass and, consequently, a reduced cost to swing [35-38]. Savings in the metabolic cost of swinging the limbs may be of high importance in terrestrial locomotion, as the swinging of limbs can account for as much as 24% of the total metabolic energy expended [39]. However, the morphology of distal limb segments, and perhaps consequently their relative mass, may be strongly influenced by functions apart from terrestrial locomotion. The morphology of distal limb segments can be specialized for functions as varied as swimming, climbing, prey capture, and digging while still being able to meet the demands of terrestrial locomotion [12,40]. It therefore remains possible that functions of the limb outside of terrestrial locomotion influence the mass of the distal segments. The scaling of limb segment masses and their potential to influence locomotor costs merits investigation.

**Figure 1:**
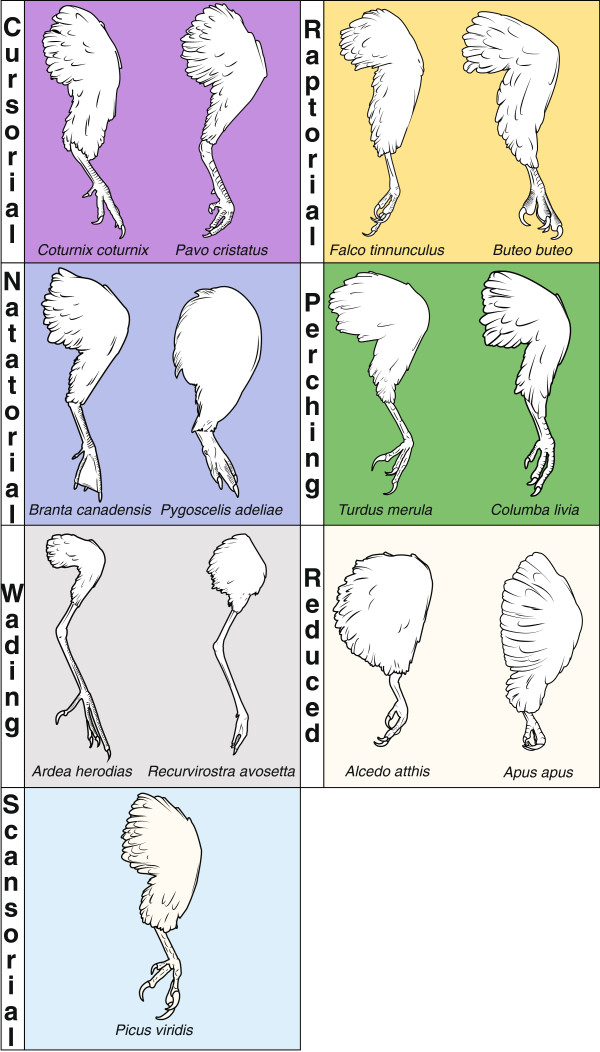
**Examples of limb specializations in neognath birds.** Examples are restricted to species sampled in the current study, and background colors for each color are associated with branch length colors in Figure 2. ‘Cursorial,’ ‘natatorial,’ and ‘scansorial’ denotes limbs specialized for running, swimming, and climbing, respectively. ‘Raptorial’ denotes limbs specialized for capturing prey. ‘Reduced’ refers to taxa possessing limbs that are relatively reduced in size and that exhibit a limited ability to walk and run.

Given their distinction as one of the most species rich amniote lineages [41,42], neognath birds are an excellent group in which to investigate how limb proportions in terms of segment masses vary with changes in body and limb size. Notably, the species comprising Neognathae possess a diversity of hindlimb functional specializations (Figure 1) [43] that allow for assessment of general scaling trends in spite of numerous limb specializations. Among avian hindlimb segments, the scaling of bone length and mass has been thoroughly studied to broaden our understanding of the influence of size in avian terrestrial locomotion. In particular, femoral and digit III length are isometric with respect to body mass, whereas the lengths of the tibiotarsus and tarosmetatarsus are positively allometric [12,14,15,23]. The positive allometry of the tibiotarsus and tarsometatarsus likely act to increase limb and stride length in larger bodied birds, though birds of a similar mass can vary greatly in limb length [44]. Bennett [45] found that in ‘non-cursorial birds’ as a group (e.g., taxa specialized for swimming, wading, perching, and climbing), limb bone lengths scale according to isometry. These differences in scaling trends among Aves as a whole [12,14,15], cursorial birds [44], and non-cursorial birds [45] suggest that functional specializations can influence the scaling trends of avian long bones relative to body mass.

Cubo & Casinos [23] found that femoral, tibiotarsal, and tarsometatarsal mass all scale with positive allometry relative to body mass (the authors did not study phalangeal mass). Thus, the skeletal contribution to segment mass is positively allometric. Yet it remains unknown whether soft tissue mass scales in parallel to bone mass. Thus a discrepancy between the scaling of bone mass and total segment mass may exist. In light of this, studying the scaling of segment mass can provide first insights into whether segment mass is tightly coupled to bone mass.

### Diversification of hindlimb morphology

**Figure 2:**
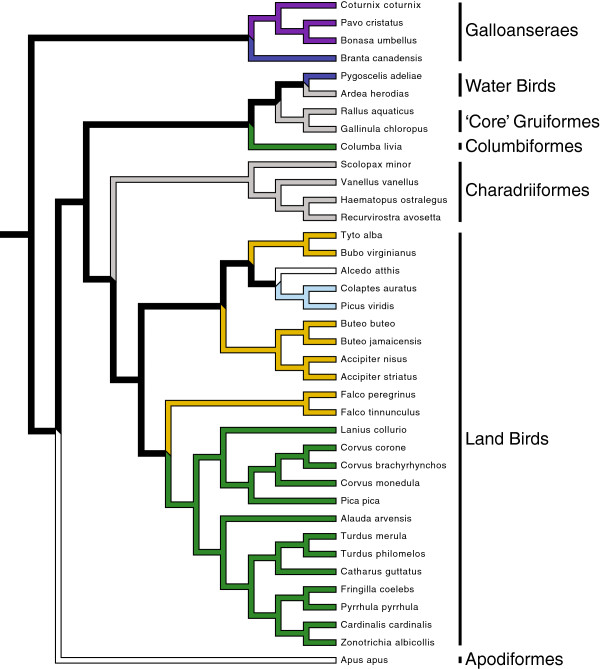
**Sampled species and their phylogenetic relationships.** Phylogeny generated from Jetz et al. [42] based upon the phylogeny of Hackett et al. [49]. Among the limb specializations sampled are cursorial (purple), perching (green), wading (grey), scansorial (light blue), natatorial (dark blue), raptorial (ochre), and aerial (white).

Given the morphological changes within the neognath forelimb for flight, the hindlimbs have often been co-opted for numerous locomotor and ecological functions, having specialized morphologies for running, climbing, and prey capture, among other functions [43] (Figures 1 and 2). Previously, it has been concluded that the dissociation of the hindlimb from the tail allowed for a diversification of hindlimb morphology (at least in terms of linear proportions) and was pivotal to the radiation of avialan birds as far back as the Early Cretaceous [6,9,46]. Thus, a diversification in hindlimb morphology appears to have been pivotal to early avian evolution and expansion into differing ecological niches. Using segment mass data, fitting of trait diversification models can reveal how changes in limb design are distributed along the branches of the phylogeny and to what extent changes in hindlimb morphology have played a role in neognath evolution [47]. For instance, trait changes occurring at a constant rate across the branches of a phylogeny can result in variation in a trait being proportional to the branches separating taxa [47] (i.e., significant phylogenetic signal). However, strong fluctuations in the rate of trait change may potentially result in phylogeny having little influence on trait values observed in species, with ecological and functional demands possibly imposing a greater influence (i.e., insignificant phylogenetic signal). Alternatively, changes in limb morphology may cluster towards the root or the tips of the tree, indicating to what degree morphological changes have contributed to early diversification of a lineage or to the terminal branches giving rise to individual species [48] (i.e., accelerations or decelerations in trait evolution).

Here I test for departures from isometry with regards to the scaling of the masses of the femoral, tibiotarsal, tarsometatarsal segments and digits relative to body mass and hindlimb length, which are measures of body size and hindlimb size, respectively. My sample of neognath birds encompasses a high diversity in terms of hindlimb morphology and function. By using such a sample, I aim to discern general trends in the scaling of segment mass in neognath birds. Negative allometry of segment masses, particularly those of distal segments would indicate changes in mass proportions beneficial for locomotor economy of larger neognaths. By using an ecologically diverse, taxonomically wide sample of neognath birds, I will also make initial inferences into how segment masses have diversified in this lineage. Through use of branch length transformation models, I will investigate models of trait change in relation to branch lengths of the phylogeny. Study of the scale effects in the mass proportions of the neognath hindlimb can serve as a platform for future work on the scaling of limb and limb segment design in birds in general and other tetrapod clades. Likewise, applying models of trait evolution to limb morphology may serve as a means for understanding how morphological changes in the locomotor system enable diversification of amniote clades.

## Materials and methods

### Sampling

Specimens of 38 neognath species (Figure 2; Table 1) were obtained from the Phyletischem Museum at Friedrich-Schiller-Universität Jena in Jena, Germany and the Field Museum of Natural History in Chicago, USA. Sampled taxa were chosen to represent major lineages and functional types within Neognathae, with neognath subclades being based upon the phylogeny of Hackett et al. [49]. Species were sampled to reflect a diverse range of hindlimb specializations, including: cursorial (running), scansorial (climbing), natatorial (swimming), raptorial (prey capturing), perching, and wading. Additionally, taxa with greatly reduced hindlimbs relative to body size and a limited ability to walk [12], such as kingfishers (*Alcedo atthis*) and swifts (*Apus apus*), were also sampled. Given that scaling trends are known to differ between taxonomic levels [50], I also examined scaling trends in the neognath subclade Land Birds (*sensu*[49]), which also contains several different functional types itself (Figure 2). Note that In spite of its name, membership in the subclade Land Birds does not denote strict or predominant terrestriality, and this group actually does not include galliform birds.

**Table 1:** **Sampled neognath taxa**, **following the taxonomy of Hackett et al.**[49]

**Species**	**N**	**Body mass (g)**	**Hindlimb length (cm)**	**Thigh mass (g)**	**Shank mass (g)**	**Pes mass (g)**	**Tars. mass (g)**	**Digit mass (g)**
**Land birds**								
*Falco tinnunculus*	1	170.0	15.9	3.92	5.52	2.45	--	--
*Falco peregrinus*^1^	1	425.9	20.7	10.60	10.90	5.60	2.60	2.90
*Corvus corone*	1	575.0	19.4	18.91	18.11	6.58	2.91	3.61
*Corvus brachyrhynchos*^1^	1	402.0	--	12.80	14.70	4.60	2.10	2.50
*Pica pica*	1	195.0	15.2	7.08	7.47	1.68	0.88	0.80
*Corvus monedula*	1	255.0	17.1	6.50	7.71	2.40	1.23	1.16
*Accipiter nisus*	1	260.0	19.7	7.15	9.15	3.07	1.80	1.26
*Accipiter striatus*^1^	1	111.6	16.0	3.00	3.90	2.20	1.20	0.90
*Buteo buteo*	2	780.0	25.1	30.88	37.81	15.16	8.71	6.42
*Buteo jamaicensis*^1^	2	2185.0	30.7	46.10	51.05	20.75	11.50	9.15
*Bubo virginianus*^1,2^	1	1028.0	30.6	44.20	59.60	25.30	12.30	13.00
*Tyto alba*	1	218.0	19.1	8.41	12.18	4.87	2.58	2.29
*Lanius collurio*	1	30.0	7.9	0.75	0.73	0.20	0.09	0.11
*Turdus merula*	2	97.5	11.1	2.50	2.43	0.59	0.27	0.32
*Turdus philomelos*	2	69.0	10.0	1.87	1.54	0.36	0.18	0.18
*Alauda arvensis*	1	40.0	--	0.88	0.77	0.20	0.08	0.11
*Fringilla coelebs*	1	18.0	6.1	0.30	0.24	0.09	0.04	0.05
*Pyrrhula pyrrhula*	1	28.0	6.5	0.44	0.36	0.13	0.06	0.07
*Cardinalis cardinalis*^1^	2	34.5	--	0.55	0.57	0.29	0.14	0.15
*Cathurus guttatus*^1^	1	29.5	9.7	0.53	0.66	0.21	0.11	0.10
*Zonotrichia albicollis*^1^	1	28.2	8.7	0.60	0.52	0.23	0.104	0.12
*Alcedo atthis*	1	33.0	5.6	0.41	0.21	0.12	0.05	0.06
*Picus viridis*	1	205.0	12.2	5.61	3.75	1.19	0.53	0.66
*Colaptes auratus*	1	135.4	9.8	3.40	2.30	0.90	0.40	0.50
**Charadriiformes**								
*Vanellus vanellus*	1	178.0	15.9	5.14	4.03	1.58	0.87	0.69
*Recurvirostra avosetta*	1	195.0	23.3	5.20	7.30	4.44	2.57	1.81
*Haematopus ostralegus*	1	433.3	20.1	15.74	10.96	4.69	--	--
*Scolopax minor*^1^	2	162.4	13.1	6.45	3.70	1.09	0.55	0.50
**Water Birds**								
*Pygoscelis adeliae*	1	4030.0	25.4	138.75	77.45	30.5	11.65	18.65
*Ardea herodias*^1^	1	2300.0	45.9	31.70	43.00	18.10	13.00	5.00
‘**Core**’ **Gruiformes**								
*Rallus aquaticus*	1	83.0	13.7	4.34	3.30	1.10	0.57	0.53
*Gallinula chloropus*	1	380.0	19.5	21.45	13.14	4.47	2.00	2.44
**Galloanseraes**								
*Bonasa umbellus*	1	564.9	20.08	21.05	13.65	3.15	1.35	1.75
*Coturnix coturnix*	1	205.0	15.2	9.39	6.84	1.87	0.87	0.96
*Pavo cristatus*^1^	1	2775.0	39.8	154.17	134.52	47.61	28.10	19.41
*Branta canadensis*^1^	1	6975.0	36.0	108.20	109.40	22.10	13.60	8.40
**Apodiformes**								
*Apus apus*	2	40.0	4.9	0.83	0.70	0.17	0.08	0.09
**Columbiformes**								
*Columba livia*	2	322.5	14.5	7.14	5.32	2.04	1.02	1.03

‘Tars’ denotes tarsometatarsus.

^1^Thigh and shank segments skinned.

^2^Body mass value from Field et al. [51].

Data were collected from specimens slated to be prepared as skeletal specimens and stored wholly intact and frozen in airtight bags in deep freezers as they awaited preparation. Though specimens were not freshly dead (i.e., < 24 hours dead), the use of airtight bags of the specimens insured against desiccation/freeze-drying. Inspection of specimens after thawing and manipulation of limb joints also prevented use of desiccated specimens.

### Data collection

**Figure 3:**
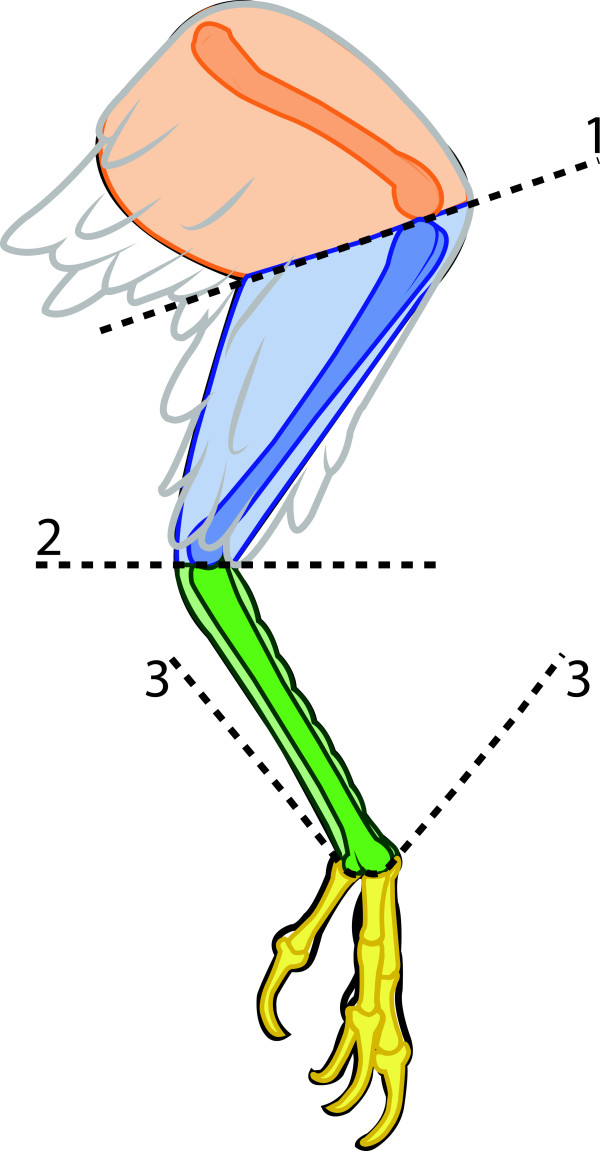
**Locations incisions to separate the limb into the following segments: thigh (orange), shank (blue), tarsometatarsal segment (green), and digits (yellow).** The mass of the pes segment (green and yellow together) was recorded prior to disarticulating the tarsometatarsal segment from the digits.

Prior to skeletonizing, each specimen’s body mass was weighed. Next, hindlimbs were dissected from the torso by carefully shaving the extrinsic muscles off the lateral face of the pelvis. The initial incisions were made along the dorsal edge of the ilia and the distal-most extreme of the pubis. To separate the limb into segments, transverse cuts were made though the knee and intertarsal joints. These cuts separated the limb into thigh, shank, and pedal segments (Figure 3). After recording the mass of the entire pes segments, an incision was made passing through the tarosometatarsal-phalangeal joints to separate the tarsometatarsal segment from the digits.

Prior to cutting the limb into segments, hindlimb length was measured in its passively flexed position [37]. To determine the passively flexed length of the limb, the limb was manually extended to its maximum and then allowed to flex on its own accord. This particular method of determining limb length was chosen given the known differences in posture between smaller and larger bodied birds [52]. As smaller bodied birds maintain a crouched, more flexed limb posture, and larger bodied birds maintain an upright, less flexed posture, measuring the passively flexed length of the limb takes into account these differences in posture and does not distort the data for small or large bodied neognaths. Note, however, that my measure of hindlimb length specifically reflects size-dependent differences in posture and is not an attempt to mimic *in vivo* limb movements or standing posture for each species.

### Statistics

Scaling relationships were assessed using Reduced Major Axis (RMA) model II bivariate regression. Prior to regression analysis, data consisting of species means were log-transformed. RMA regressions were my chosen method of analysis, as Model II regressions assume that both x and y variables contain some degree of error (either measurement errors and/or biological variation) and are not independent in the strict sense [53,54]. Moreover, RMA regressions are ideal for testing slope values against null model predicted values [55]. Additionally, to identify how segment masses co-diversified against body mass and hindlimb length, I also separately performed phylogenetic generalized least squares (PGLS) regressions (see below).

Under isometric scaling, segment mass should scale as (body mass)^1.0^ and (hindlimb length)^3.0^[56]. Log transformation of the data changes the scaling relationship from its normal power function expression of y = a(x)^b^ into a linear linear function: log(y) = log(a) + log(b)(x). Thus, according to isometry, the regression slope should be 1.0 and 3.0 when scaling against body mass or hindlimb length, respectively [56,57]. To test for departures from isometry, two methods were used. The first method was an F-test to test whether the empirical value significantly deviates from isometry’s prediction, with deviations being significant if *P* < 0.05. The second method, utilizing effect size statistics [58], employed 95% confidence intervals for the regression slope. If slope confidence limits exclude the predicted value, then isometry was rejected. F-tests were performed and confidence intervals were calculated in R version 2.15.1 [59] using the module SMATR [55]. Through a combination of effect size and significance value based statistics, an increase in Type I error rates due to repeated testing bias is avoided, as is a decrease in statistical power due to Bonferroni corrections [60].

For both the entire Neognath sample and Land Bird subsample, differences in slope and intercept (i.e., ‘elevation’ following the terminology of Warton et al. [55]) of RMA regressions were identified by using common slope tests and Wald’s test, respectively. If *P* < 0.05, then differences in regression slope and intercept were considered significant. Tests for common slope and intercept were performed in the R module SMATR [55].

### Comparative methods

Species data is not independent due to hierarchically structured phylogenetic relationships among species. As such, conventional statistical methods are not suited for estimating evolutionary models of trait evolution or inferring the evolutionary processes that produce empirical trait values [61]. For segment masses, body mass, and hindlimb length, I tested two models of trait evolution by transforming branch lengths with Pagel’s λ and Pagel’s δ [48].

λ is a branch length transformation that models the dependence of observed trait variation on phylogenetic relationships of a given tree [48,62]. It should be noted that λ is a often used as a direct measure of phylogenetic signal – the tendency for increased phenotypic similarity with increasing phylogenetic relatedness [62,63] – within each trait. A multiplicative factor of a tree’s internal branches, λ of 0.0 indicates a complete absence of phylogenetic signal and that traits evolved independently among the individual sampled taxa; in contrast, a λ of 1.0 indicates that traits evolved by constant-rate Brownian motion along the branches of the tree [48,64]. In theory, a value of 1.0 indicates that rates of trait change have remained constant across the tree; however, inferring a relationship between phylogenetic signal and rates of trait change is highly problematic and should be avoided [62].

*δ* is a branch length transformation that models whether rates of trait change are greater towards the root or the tips of the tree [48,65], acting as a multiplicative factor of both shared and internal branches lengths on the tree. δ > 1.0 indicates that more recent evolution within a clade has had a greater influence on trait diversification. In contrast, δ < 1.0 indicates that early evolution within a clade has a had a greater influence upon trait diversification. δ = 1.0 indicates that a trait diversified under a model of Brownian motion and the branch lengths remain unchanged. It is important to note that δ represents only a monotypic increase or decrease in rates of trait change across the tree. In all likelihood though, rates of trait change differ amongst the different branches of the tree, and there are existing methods to check for such differing rates (e.g., auteur: [66]). However, given my sample size, my data is poorly suited to methods such as auteur, which is ideally suited by datasets and phylogenies with at least ~ 60 taxa. In spite of this, using a δ transform can reveal whether rates of change in segment masses are not monotypic along the tree.

To test whether trait diversification in terms of both λ and δ departed from a Brownian motion model, 95% confidence intervals were generated for both of these parameters for each trait studied, with an exclusion of 1.0 indicating a departure from Brownian motion. λ , δ, and accompanying confidence limits were estimated using the module pmc (Phylogenetic Monte Carlo; [67]) in R.

Furthermore, the fit of each model was compared using a Monte Carlo-based method in pmc. First, the likelihood ratio was calculated as the difference between the log likelihood of observing the data under maximum likelihood models of λ and δ. Then under the λ model, a given trait was simulated as evolving along the specified phylogeny over 1000 iterations. For each iteration, a λ and δ model were fit to the data and the likelihood ratio between the two fits was calculated. From the 1000 iterations, a distribution of likelihood ratios was calculated with a 95% confidence interval. If the confidence interval excluded the observed likelihood ratio, here acting as a critical value, then the λ model is rejected (i.e., the observed likelihood ratio is not the result of applying both the λ and δ models to a trait that has evolved in line with a λ model). This procedure is then repeated simulating a given trait as evolving under a δ model – a likelihood ratio distribution and accompanying 95% confidence interval are generated by applying the two models to simulated data evolving under Pagel’s δ. As in the test of Pagel’s λ, the observed likelihood ratio is used as a critical value in combination with the confidence limits to test this second model of trait evolution. For a more detailed explanation of the pmc method, see Boettiger et al. [67]. Given that the pmc module only allows pair-wise comparisons of models, I compared each model to a Brownian motion model using confidence limits for λ and δ as described above, whereas to directly to compare these two models and test their fit of the data, I used the Monte Carlo-based methods in pmc.

I also applied the models of Pagel’s λ and δ to residuals from bivariate generalized least squares regressions of segment masses against body mass and hindlimb length. Even though individual traits may follow a given evolutionary model, it does not necessarily guarantee that the traits have co-diversified under such a model [68]. To determine if segment mass traits have co-diversified with measures of body and limb size in line with the two trait diversification models, I generated 95% confidence intervals for λ and δ and used the Monte Carlo based method of Boettiger and colleagues [67] to test the fit of these models to regression residuals [68]. In addition to Monte Carlo-based methods of model fit, I also repeated each bivariate regression for the entire neognath sample as a Phylogenetic Generalized Least Squares (PGLS) regression. Performing PGLS regressions can illuminate whether the co-diversificaiton of segment masses and measures of size has been either allometric or isometric.

To test for diversification models for each trait, I used the phylogeny of Jetz et al. [42] with internal nodes based upon the phylogeny of Hackett et al. [49]. A tree consisting of the sampled species was generated using the website http://birdtree.org. Branch lengths were based upon divergence times in absolute time.

## Results

### Body mass scaling

**Figure 4:**
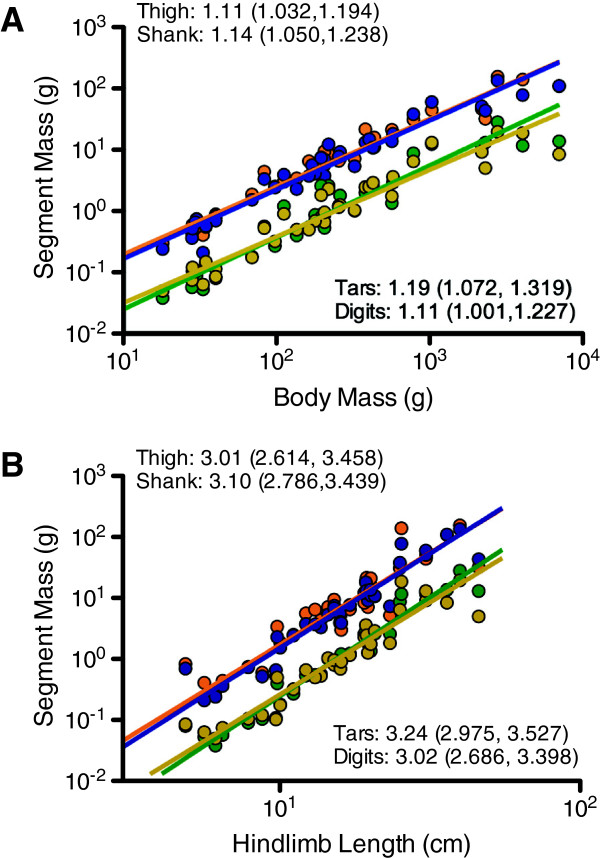
**Scaling of thigh (orange), shank (blue), tarsometatarsal segment (green), and digit (yellow) masses for the entire neognath sample.** The upper plot **A** depicts scaling relationships relative to body mass; the lower plot **B** depicts scaling relationships relative to hindlimb length. In addition to the slope and confidence limits provided in the plots, the results of F-tests testing for departures from isometry are listed in Table 2.

For the entire neognath sample, all segment masses scale with positive allometry relative to body mass (Figure 4A and Table 2). Slopes range between 1.11 (thigh segment and digits) to 1.19 (tarsometatarsal segment). A common slope test finds that segments do not differ in slope (P = 0.8279); however, the segments do differ in regression intercept/elevation (P < 0.0001). Post hoc tests reveal that the thigh and shank segments do not significantly differ in intercepts (P = 0.3657); likewise, the tarsometatarsal segments and digits do not differ in intercept (P = 0.9250). However, the two proximal segments (thigh and shank segments) significantly differ in intercept from the two distal segments (tarsometatarsal segment and digits) (P < 0.05).

**Table 2:** Results of regressions of segment masses against body mass and hindlimb length for the entire neognath sample

**Trait**	**N**	**Int.**	**95% C.I.**	**Slope**	**95% C.I.**	**R**^**2**^	***P***_**GS**_
Body mass scaling							
Thigh mass	38	-1.82	-2.018, -1.625	1.11	1.032, 1.194	0.9530	**0.0065**
Shank mass	38	-1.93	-2.155, -1.700	1.14	1.050, 1.238	0.9402	**0.0027**
Pes mass	38	-2.41	-2.677, -2.135	1.15	1.044, 1.269	0.9167	**0.0058**
Tars. mass	36	-2.81	-3.105, -2.510	1.19	1.072, 1.319	0.9113	**0.0017**
Digit mass	36	-2.63	-2.898, -2.354	1.11	1.001, 1.227	0.9146	**0.0479**
Hindlimb length scaling							
Thigh mass	35	-2.77	-3.287, -2.263	3.01	2.614, 3.458	0.8430	0.9757
Shank mass	35	-2.92	-3.317, -2.525	3.10	2.786, 3.439	0.9113	0.5501
Pes mass	35	-3.42	-3.779, -3.065	3.14	2.855, 3.444	0.9298	0.3449
Tars. mass	33	-3.49	-4.182, -3.514	3.24	2.975, 3.527	0.9459	0.0754
Digit mass	33	-3.60	-4.033, -3.171	3.02	2.686, 3.398	0.8966	0.9057

‘Int.’ and ‘Tars.’ denote ‘intercept’ and ‘tarsometatarsus,’ respectively. *P*_GS_ are the results of F-tests testing for departures from the null model.

Bold values of P_GS_ indicate departures from isometry’s prediction.

**Figure 5:**
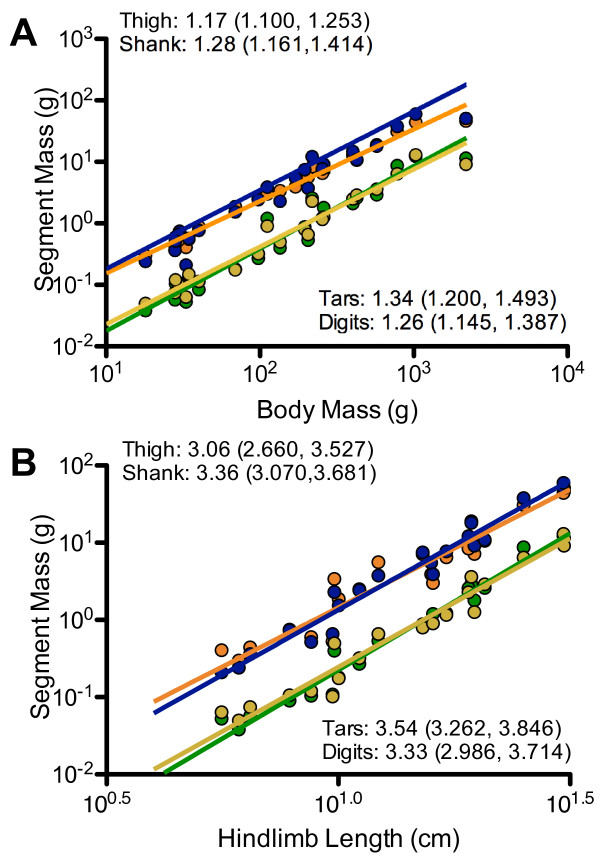
**Scaling of thigh (orange), shank (blue), tarsometatarsal segment (green), and digit (yellow) masses for the Land Bird subsample.** The upper plot **(A)** depicts scaling relationships relative to body mass, whereas the lower plot **(B)** depicts scaling relationships relative to hindlimb length. In addition to the slope and confidence limits provided in the plots, the results of F-tests testing for departures from isometry are listed in Table 3.

With regards to the Land Bird subsample, all segment masses scale with positive allometry (Figure 5A and Table 3). Slopes range from 1.17 (thigh segment) to 1.34 (tarsometatarsal segment). The segments do not differ in slope (*P* = 0.1877). For the entire neognath sample, segment masses significantly differ in intercept (*P* < 0.0001). As is the case for the neognath sample, the proximal-most and distal-most pairs of segments differ in intercept as indicated by post hoc tests (*P* < 0.05). However, the two proximal-most segments (thigh and shank segments) do not differ in intercept (*P* = 0.9877), and the two distal-most segments (tarsometatarsal segment and digits) likewise do not differ in intercept (*P* = 0.7214).

**Table 3:** Results of regressions of segment masses against body mass and hindlimb length for the Land Bird subsample

**Trait**	**N**	**Int.**	**95% C.I.**	**Slope**	**95% C.I.**	**R**^**2**^	***P***_**GS**_
Body mass scaling							
Thigh mass	24	-1.98	-2.143, -1.807	1.17	1.100, 1.253	0.9783	**< 0.0001**
Shank mass	24	-2.02	-2.480, -1.925	1.28	1.161, 1.414	0.9503	**< 0.0001**
Pes mass	24	-2.69	-2.981, -2.398	1.30	1.177, 1.442	0.9472	**< 0.0001**
Tars. mass	23	-3.09	-3.412, -2.767	1.34	1.200, 1.493	0.9415	**< 0.0001**
Digit mass	23	-2.90	-3.170, -2.639	1.26	1.145, 1.387	0.9553	**< 0.0001**
Hindlimb length scaling							
Thigh mass	21	-2.90	-3.393, -2.398	3.06	2.660, 3.527	0.9131	0.7624
Shank mass	21	-3.23	-3.582, -2.881	3.36	3.070, 3.681	0.9641	**0.0167**
Pes mass	21	-3.75	-4.110, -3.399	3.44	3.140, 3.760	0.9647	**0.0051**
Tars. mass	20	-4.19	-4.520, -3.852	3.54	3.262, 3.846	0.9723	**0.0005**
Digit mass	20	-3.94	-4.354, -3.521	3.33	2.986, 3.714	0.9513	0.0597

‘Int.’ and ‘Tars.’ denote ‘intercept’ and ‘tarsometatarsus,’ respectively. *P*_GS_ are the results of F-tests testing for departures from the null model.

Bold values of PGS indicate departures from isometry’s prediction.

For all regressions, the range of confidence limits and the results of F-tests do not disagree when testing for departures from geometric similarity (Tables 2 and 3).

### Limb length scaling

Regarding the entire neognath sample, segment masses scale isometrically with hindlimb length (Figure 4B and Table 2). Slopes ranged between 3.01 (thigh segment) and 3.24 (tarsometatarsal segment). Among the limb segments, the slopes do not differ (*P* = 0.8433); however, as when scaling against body mass, the proximal and distal pairs of limb segments differ in intercept (*P* < 0.0001). Yet, as is the case scaling when against body mass, the segments comprising each pair do not differ in slope from one another (*P* > 0.05).

For the Land Bird subsample, segment masses scale isometrically or with positive allometry when scaled against hindlimb length (Figure 5B and Table 3). Slopes range from 3.06 to 3.54. Thigh segment and digit mass are isometric with hindlimb length, not significantly differing from a slope of 3.0, whereas shank segment and tarsometatarsal segment mass are both positively allometric (Table 3). However, directly comparing slopes across limb segments finds no significant difference in slope (*P* = 0.4780). Post hoc tests uncover that the thigh and shank segments significantly differ in intercept from the tarsometatarsal segment and digits (*P* < 0.0001). However, the thigh and shank segments do not differ in slope (*P* = 0.9950), just as the tarsometatarsal segment and digits do not (*P* = 0.8396).

For all regressions, the range of confidence limits and the results of F-tests agree when testing for departures from geometric similarity (Tables 2 and 3).

### Trait diversification

For each individual trait examined, estimates of both λ and δ suffer from wide confidence limits, indicating a high degree of uncertainty in parameter estimation (Table 4). However, confidence limits for all traits exclude a value of 0.0, revealing a significant phylogenetic influence on trait variation. Regarding Brownian motion, λ and δ both have confidence limits including a value of 1.0, indicating that Brownian motion evolution cannot be rejected. Log likelihood ratio confidence intervals for the model itself are wide and encompass the observed likelihood ratios (i.e., critical values) for all traits. Consequently, neither λ or δ can be rejected as a model of trait diversification.

**Table 4:** Models of trait diversification fit to each trait

**Trait**	**N**	**λ**	**λ C. L.**	**L**	**Model C.L.**	**δ**	**δ C.L.**	**L**	**Model C.L.**	**R**
Body mass	38	0.95	0.615, 1.000	-29.47	-3.243, 3.059	1.55	0.834, 3.000	-29.69	-3.785, 1.154	-0.44
Hindlimb length	38	1.00	0.773, 1.000	7.97	-0.679, 2.822	1.08	0.574, 3.000	7.98	-3.105, 0.712	0.02
Thigh mass	38	1.00	0.855, 1.000	-31.56	-0.749, 3.274	1.33	0.719, 3.000	-31.44	-3.785, 0.802	0.24
Shank mass	38	1.00	0.821, 1.000	-31.85	-0.694, 3.160	1.13	0.625, 3.000	-31.83	-3.388, 0.897	0.04
Pes mass	38	1.00	0.807, 1.000	-32.46	-0.651, 3.288	1.27	0.706, 3.000	-32.38	-3.596, 0.867	0.16
Tars. mass	36	1.00	0.802, 1.000	-32.78	-0.672, 3.365	1.29	0.746, 3.000	-32.70	-3.221, 0.788	0.16
Digit mass	36	1.00	0.831, 1.000	-30.95	-0.664, 3.237	1.42	0.723, 3.000	-30.78	-3.499, 0.766	0.34

λ and δ C.L. denotes 95% confidence limits for estimates of these parameters, L denotes log likelihood values for each model, and Model C.L. denotes the 95% confidence limits of likelihood ratios used to test each model. R denotes the observed likelihood ratio that is used as a critical value to test each model via exclusion from likelihood ratio confidence limits.

Regarding the co-diversification of segment masses alongside body size, for the thigh, shank, and pes, likelihood ratio confidence limits indicate that λ is a better descriptor of trait co-diversification than δ (Table 5). This is in spite of the wide confidence limits for λ indicating the uncertainty and difficulty of fitting this parameter to the data and the ability of δ estimates to reject both a lack of phylogenetic influence and Brownian motion through δ confidence limits excluding a value of 0.0 and 1.0, respectively. For the tarsometatarsal segment and digits, both λ and δ reject a lack of phylogenetic influence and indicate trait evolution by Brownian motion. However, neither model can be rejected in favor of the other (Table 5).

**Table 5:** **Models of trait co**-**diversification fit to each trait alongside body mass or hindlimb length**

		**Model λ**				**Model δ**				
**Trait**	**N**	**λ**	**λ C. L.**	**L**	**Model C.L.**	**δ**	**δ C.L.**	**L**	**Model C.L.**	**R**
Body mass scaling										
Thigh mass	38	0.82	0.200, 1.000	19.63	-8.727, 2.935	3.00	1.606, 3.000	16.57	-5.071, 3.182	-6.12
Shank mass	38	0.80	0.091, 1.000	13.25	-9.149, 3.038	3.00	1.645, 3.000	9.98	-5.074, 2.586	-6.54
Pes mass	38	0.76	0.000, 1.000	6.44	-10.816, 2.840	3.00	1.598, 3.000	3.86	-4.755, 3.067	-5.16
Tars. mass	36	1.00	0.821, 1.000	-32.78	-0.932, 3.064	1.29	0.675, 3.000	-32.70	-3.670, 0.952	0.16
Digit mass	36	1.00	0.796, 1.000	-30.95	-0.618, 3.123	1.42	0.785, 3.000	-30.78	-3.514, 1.226	0.34
Hindlimb length scaling										
Thigh mass	35	0.94	0.596, 1.000	-4.24	-2.489, 2.840	2.96	1.492, 3.000	-2.61	-4.072, 2.831	3.26
Shank mass	35	0.94	0.696, 1.000	4.99	-2.557, 3.085	2.72	1.507, 3.000	5.96	-4.242, 2.777	1.94
Pes mass	35	0.77	0.000, 1.000	7.96	-7.367, 2.382	3.00	1.539, 3.000	8.68	-4.146, 2.845	1.44
Tars. mass	33	1.00	0.732, 1.000	-32.42	-0.674, 2.939	1.56	0.821, 3.000	-32.16	-3.223, 0.855	0.52
Digit mass	33	1.00	0.762, 1.000	-30.84	-0.528, 2.984	1.72	0.883, 3.000	-30.45	-3.682, 1.341	0.78

λ and δ C.L. denotes 95% confidence limits for estimates of these parameters, L denotes log likelihood values for each model, and Model C.L. denotes the 95% confidence limits of likelihood ratios used to test each model. R denotes the observed likelihood ratio that is used as a critical value to test each model via exclusion from likelihood ratio confidence limits.

Regarding the co-diversification of segment masses alongside limb length, likelihood ratio confidence limits do not reject either model (Table 5). Both λ (with the exception of the thigh) and δ indicate that phylogeny significantly influences variation in residuals with confidence intervals excluding a value of 0.0. Neither λ and δ can reject a model of Brownian motion for the co-diversification of segment masses with limb length, as all confidence intervals include a value of 1.0.

PGLS regressions indicate that segment masses have co-diversified isometrically alongside body mass and hindlimb length (Table 6).

**Table 6:** Results of PGLS regressions

**Trait**	**N**	**Int.**	**95%C.I.**	**Slope**	**95%C.I.**	**L**
Body mass scaling						
Thigh mass	38	-1.56	-1.898, 1.213	1.00	0.892, 1.100	14.86
Shank mass	38	-1.59	-1.991, -1.182	0.99	0.863, 1.109	8.53
Pes mass	38	-2.05	-2.527, -1.576	0.98	0.834, 1.123	2.37
Tars. mass	36	-2.41	-2.943, -1.876	1.00	0.842, 1.167	-1.70
Digit mass	36	-2.35	-2.823, -1.884	0.97	0.826, 1.112	2.87
Hindlimb length scaling						
Thigh mass	35	-2.38	-3.091, -1.663	2.75	2.246, 3.252	-4.31
Shank mass	35	-2.65	-3.202, -2.097	2.92	2.530, 3.309	4.66
Pes mass	35	-3.21	-3.734, -2.682	2.98	2.609, 3.350	6.41
Tars. mass	33	-3.64	-4.141, -3.147	3.09	2.742, 3.443	7.83
Digit mass	33	-3.37	-3.993, -2.755	2.85	2.411, 3.283	0.61

‘95% C.I.’ refers to the 95% confidence interval for the slope and intercept (Int.). ‘L’ denotes the log likelihood value.

## Discussion

### Scale effects

All segment masses scale with positive allometry relative to body mass, whereas they scale isometrically relative to hindlimb length. Thus, limb segment masses do not scale with negative allometry or with increasingly lower scaling exponents distally along the limb, which would reduce the cost of swinging the limbs in larger neognath species. Rather, scale effects of individual hindlimb segments parallel the scaling of whole hindlimb mass relative to body mass (i.e., positive allometry) and hindlimb length (i.e., isometry) [37]. In light of these results, the scale effects in the hindlimb’s mass proportions do not afford a lowered cost of swinging the limbs in neognath birds with respect to increasing size. However, the differences in regression elevation indicate that the pes and its constituent segments have less mass than the more proximal segments (in absolute terms) for a given body mass or limb length (Figures 4 and 5). Consequently, absolute differences in mass between proximal and distal segments result in limbs with a lowered cost of swinging compared to limbs with a more even distribution of mass between proximal and distal segments.

The scaling of segment masses differs from the scaling of segment lengths, which are determined by limb bone lengths. Amongst the limb segments, only the lengths of the tibiotarsus and tarsometatarsus scale with positive allometry relative to body mass; the lengths of the femur and digit III in contrast scale with isometry [12,14,15,44,45]. Thus, the scaling of limb segment mass is not necessarily tied to the scaling of limb segment length. In contrast – and perhaps not surprisingly – scale effects in segment mass may be more strongly tied to the scaling of hindlimb bone mass. Much like the masses of their respective segments, the masses of the femur, tibiotarsus, and tarsometatarsus all scale with positive allometry relative to body mass [23]. The positive allometry of tibiotarsal and tarsometatarsal mass is likely due to the relatively greater lengths of these long bones in larger avian species, whereas the positive allometry of femoral mass is likely due to the relatively greater femoral robusticity in larger avian species [44,69]. It also worth noting that cross-sectional area and second moment of area of these three elements all scale with positive allometry [14,70], which also likely contributes to the positive allometry of bone mass and, consequently, segment mass. However, the allometry present in the second moment of area is in part due to distribution of bone tissue about the cross-section’s neutral axis [14]. With regards to the digits, aside from data on the length of digit III [15] or the longest digit [44,45] and total digit mass (current study), scale effects in digit morphology remain unexplored (though see Pike & Maitland [71] for scale effects in claw shape). Given that many functional specializations occur in the pes – such as webbed feet and raptorial claws – future studies of scale effects in segment masses should investigate scale effects both within and across individual functional groups. However analysis of scaling trends within individual functional groups requires larger sample sizes than included in this study and must wait until subsequent studies with higher within-group sampling.

With regards to how muscle mass may contribute to the scaling of segment mass, the picture is somewhat murkier. There is no available data on how total hindlimb muscle mass scales against body or hindlimb length. However, the masses of the biceps femoris group, iliotibialis, femorotibialis, gastrocnemius, and digital flexors all tend to scale isometrically with body mass [44] or with slight positive allometry [45]. If the isometry between muscle mass and body mass is characteristic of the remaining muscles of the hindlimb, then it would indicate that the positive allometry of the mass of the muscled segments (e.g., the thigh and shanks) is due principally to the scaling of bone mass. It should be noted though that the studies of Maloiy et al. [44] and Bennett [45] used functionally based (e.g., cursorial/non-cursorial birds) samples of Aves, and the results of their study may not necessarily reflect scaling patterns for a more inclusive species sampling.

With specific regards to the mass of the pes and its constituent segments, bone mass is almost certainly the primary determinant of segment mass. Given that the flexors and extensors of the intertarsal joint and digits are concentrated on the thigh and shanks segments [72-74], the pedal segments are comprised of predominantly bone, tendon, and integument. In some species, digital extensors are weakly developed [75], and in these taxa these muscles might make minor – though significant – contributions to pedal mass. The overall concentration of muscle mass on the thigh and shank segments likely underlies differences in regression elevation between the more proximal (thigh and shank) and more distal segments (pes, tarsometatarsal segment, and digits) (Figures 4 and 5).

**Figure 6:**
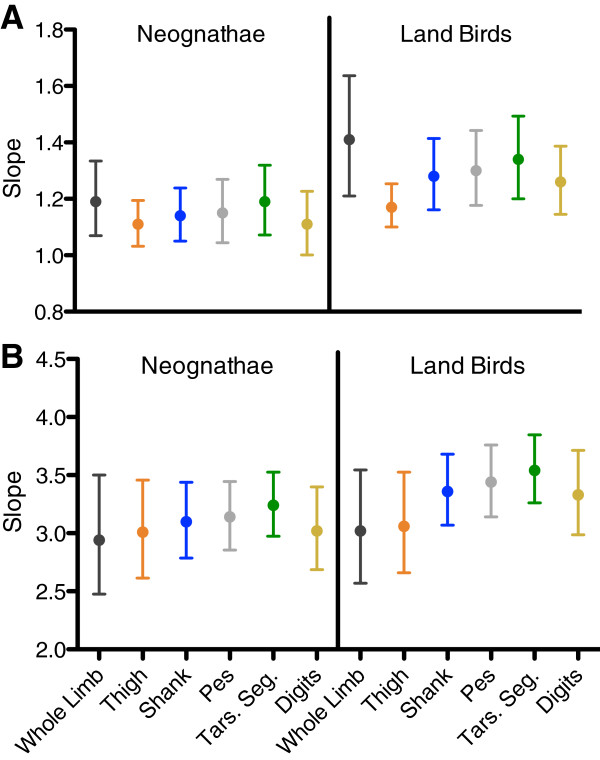
**Slopes and confidence intervals for whole hindlimbs and individual hindlimb segments. A** depicts slopes for body mass scaling, whereas **B** depicts slopes for hindlimb length scaling. Regression slopes and confidence limits for the scaling of whole limb mass are from Kilbourne [37].

Regarding scale effects in the subclade Land Birds, scale effects of segment masses vs. body mass parallel those for the entire sample of Neognathae, exhibiting positive allometry (Table 3). However, when scaling against hindlimb length, segment mass is positively allometric for the shank segment and pes. Notably when separating the pes into the tarsometatarsal segment and digits, only the mass of the former is positively allometric. The positive allometry of the shank and tarsometatarsal segments appears to be at odds with the scaling of whole hindlimb mass relative to hindlimb length, which is isometric [37]. However, comparing the slopes for whole hindlimb and individual segment mass scaled against hindlimb length (Figure 6B) reveals that in spite of the high slope estimates for the shank, pes, and tarsometatarsal segments, the confidence limits for these segments overlap with those for the whole hindlimb. The wide slope confidence limits for Land Birds is likely due to the smaller subsample size (N = 24). As a consequence, to more clearly discern how scale effects in individual hindlimb segments contribute to the scaling of overall hindlimb mass in Land Birds relative to their hindlimb length, greater sample sizes are needed.

Regarding scale effects as segment masses co-diversified with body and limb size, segment mass is positively allometric with body mass and isometric with body mass and hindlimb length (Table 6). Furthermore, inspection of the confidence intervals for the intercept reveal that, as in the raw regressions, the two proximal-most segments differ in their mass from the two distal-most segments. It thus appears that the between species differences in segment mass scale effects (i.e., raw regression results) only partly reflect how segment masses have co-diversified with body and limb size.

### Whole limb mass distribution

Like individual limb segment masses, whole hindlimb mass is positively allometric alongside body mass and isometric alongside hindlimb length [37] (Figure 6). Thus, the scaling of total limb mass is not the result of conflicting scaling trends among individual limb segments. The hindlimb’s mass distribution – as reflected by the hindlimb’s center of mass position and radius of gyration– is also positively allometric with body mass [37]. However, the positive allometry of the hindlimb’s mass distribution is not due to more distal limb segments possessing greater allometric exponents, given the lack of differing scale effects among the hindlimb’s comprising segments (Figures 4, 5 and 6). Instead, the positive allometry of mass distribution traits is likely due to the scale effects in hindlimb posture. Larger-bodied birds have a more upright posture affording the extensor muscles a greater mechanical advantage across their joints; in contrast smaller-bodied birds have a more crouched limb posture [45,52] (though see Ref. [76]). As a result of these size-related limb postures, the mass of the distal limb segments is extended farther from the hindlimb’s pivot, and, as a consequence, the hindlimb’s center of mass shifts distally with increasing body size. It should be noted also that the measure of hindlimb length used in Kilbourne [37] specifically reflected the postural differences between small and large-bodied neognath species. Though not a significant departure from isometry’s predicted exponent (1/3), the allometric exponent relating the scaling of hindlimb length to body mass is higher than the prediction (0.41 from Ref. [37]; 0.37 when reanalyzed with current sample, *P*_F-test_ = 0.1837). As the masses of hindlimb segments and the hindlimb mass distribution are all isometric with hindlimb length, it seems all the more plausible that postural differences between smaller and larger bodied neognaths underlie how the hindlimb’s mass distribution traits scale with body mass.

### Functional limitations

Negative allometry of limb segment masses would be beneifical for relatively lower costs, given that relatively less mass would need to be accelerated to swing the limb [37,38]. Likewise, negative allometry of distal limb segment masses would shift the limb’s center of mass proximally along the limb, also resulting in a relative reduction in the cost of swinging the limb [37,38]. However, in spite of the potential benefits, I found that limb segment masses scale either with positive allometry (relative to body mass) or isometry (relative to hindlimb length). The mass of body segments may be minimized in neognath species in order to minimize the cost of flight. Birds possess a number of traits that can contribute to a lowered metabolic cost of flight, including smaller body masses [77] (though see [78]), pneumatized bones [79-81], and long bones with a more efficient distribution of bone tissue about their cross-section [14,82] (though some of these traits could be exaptations enabling flight). Thus, neognaths and other birds having hindlimb segments of minimal mass is not implausible; however, isometry or negative allometry of segment mass could result in larger-bodied birds having limbs with too little mass to withstand the mechanical loads occurring not only during terrestrial locomotion but also in other functions, such as prey capture, swimming, or climbing. Conversely, negative allometry or isometry of segment masses could result in small-bodied bird having hindlimbs of greater mass, which could increase the metabolic cost of flight.

The notion that negative allometry of segment mass – particularly bone mass – may result in structurally weak limbs coincides with how bone flexural modulus scales with body mass. Among avian long bones, flexural modulus, the resistance to bending owing to both a bone’s structure and material, decreases with increasing body mass [83]. Additionally, avian long bones are not optimized to be of minimum mass. In a survey of long bone cross-sections within amniotes, Currey & Alexander [82] found that the greater minimization of bone mass in birds may result in long bones more prone to mechanical failure due to the ‘rough-and tumble’ lives of birds. Given that the predominant tissue of the distal limbs segments is bone, it seems highly possible that the negative allometry of segment masses may render the distal limb more susceptible to mechanical failure.

Alternatively, negative allometry of hindlimb segment masses may not be pivotal to neognath locomotion in light of their ability of flight. Notably flight is a cheaper means of locomotion than walking or running to cover long distances [84], though it is highly costly on a basis of per unit time [85].

### Implications for terrestrial locomotion

The lack of negative allometry of segments masses may act to hamper the terrestrial locomotor ability of larger-sized neognaths by result of limbs that are costly to swing relative to body and limb size [35]. Consequently, larger neognaths may be restricted in how quickly they can oscillate their limbs during terrestrial locomotion. In an examination of scale effects in avian terrestrial locomotion, Gatesy & Biewener [52] found that in larger avian species stride frequency increases with speed at a shallower rate than in smaller avian species, whereas stride length increases at a steeper rate with speed in larger species. Comparing species locomoting at their top speed on a treadmill, the authors found that stride frequency decreases alongside body mass, being proportional to (body mass)^-0.18^. Though this exponent is greater than the predicted exponent for isometric scaling (-1/3; [56]), it must be noted that stride frequency still overall decreases relative to increasing body size. In contrast to stride frequency, stride length for birds locomoting at their top speed increases alongside body mass, scaling as (body mass)^0.39^[52] and well above isometry’s predicted exponent of 1/3 [56]. Thus it seems that larger-bodied neognaths may ameliorate any detrimental consequences of scale effects in segment mass by favoring longer strides and relatively lower limb oscillations (i.e., stride frequencies). It should be noted that terrestrially locomoting birds also tend to increase speed by predominantly lowering stance duration. In contrast, swing duration remains invariant or undergoes only minor decreases with increasing speed not only in birds [86-95] but also in mammals [96-106]. The limiting factor on decreasing swing duration could likely be the mass and moment of inertia of the limb and its segments.

Additionally, larger bodied birds may try to allay negative consequences of segment mass scaling by changing their hindlimb kinematics relative to smaller bodied birds. Applying leg weights to the tarsometatarsal segments of running turkeys (*Meleagris gallopavo*) and guinea fowl (*Numida meleagris*) has been found to elicit a kinematic response, such as smaller limb segment excursion angles and/or longer swing durations, in light of increased energy expenditure [107,108]. Thus, in response to limb segments with relatively greater mass, especially those distal on the limb, larger birds may differ in their hindlimb kinematics relative to smaller birds by decreasing limb segment excursions or increasing joint flexion during swing phase. However, to test this hypothesis, detailed data on hindlimb joint kinematics are needed for a sample of birds diverse in both body size and limb function and locomoting over a range of speeds.

### Trait diversification

Likely owing to limited sample size [67,109], the two models of trait diversification were plagued with wide confidence intervals, indicating that caution is needed when interpreting these results. Though λ is fairly robust to increasing species sample size [110], it is highly unlikely that my limited sample (N = 38) fully reflects and encapsulates trait evolution within Neognathae, especially given this clade’s high species richness and complex evolutionary history (~10,000 species [41,42]). However, by sampling a diverse assemblage of limb specializations, I sought to highlight the role of species poor lineages with distinct hindlimb morphologies in neognath diversification.

With few exceptions, confidence intervals for λ and δ indicate that phylogeny influences variation in segment masses and scale effects by exclusion of a value of 0.0 (Tables 4 and 5). However, for both individual traits and hindlimb regression residuals, neither model for segment diversification could be rejected, highlighting the uncertainty in the data (Tables 4 and 5). Even when δ could be rejected for the co-diversification of thigh, shank, and pes mass alongside body mass, λ exhibits wide confidence limits nearly spanning bounds upon this parameter (0.0,1.0).

Though sample size likely plays a factor in these results, it is also probable that deep divergences within the phylogeny of my sampled taxa are an additional factor [67]. Examining node ages from my phylogeny reveals that the major lineages constituting my sampled taxa diverged tens of millions of years ago, such as Galloanseraes (109.5 mya), Land Birds (82.1 mya), and Apodiformes (82.9 mya) (divergence times from Jetz et al. [42]). As diversification events occur farther back in the past, the less information is retained in the tree. Given the species richness of neognaths, it is also a distinct possibility that phylogenetic signal varies across the branches of the tree or that rates of evolution do not increase or decrease linearly or monotypically. Furthermore, a lack of data from fossil taxa [111] may also increase the difficulty of identifying a model of trait evolution for hindlimb segment masses in neognaths. Ultimately, as the quality of the data does not provide enough power to distinguish between models, let alone reject either model or both, any interpretations of parameter estimates should be treated with extreme caution without greater sampling of taxa.

### Linear vs. mass proportions

With regards to the linear proportions in the avian hindlimb, variation appears to be constrained by embryonic development patterning and postnatal functional demands, both of which limit the variation of zeugopodal (i.e., tibiotarsus) length. Proximo-distal patterning of the amniote limb through activation-inhibition dynamics results in a trade-off in length between the stylopod and autopod, whereas the zeugopod exhibits reduced variation, being approximately 1/3 of total limb segment length [112]. Thus, through developmental pathways, there is decreased variation in the ‘middle segment’ of not only birds but also mammals [5,15]. However, factors other than activation-inhibition dynamics, such as postnatal growth and/or functional specializations may promote increased variation in zeugopod length. In spite of the apparent influence of developmental mechanisms, it should be noted that the reduced variation in zeugopod length still likely confers a biomechanical advantage to amniotes [5,15,113]. Yet does the variation in relative proportions of segments lengths apply as well to the mass proportions of the avian hindlimb?

**Figure 7:**
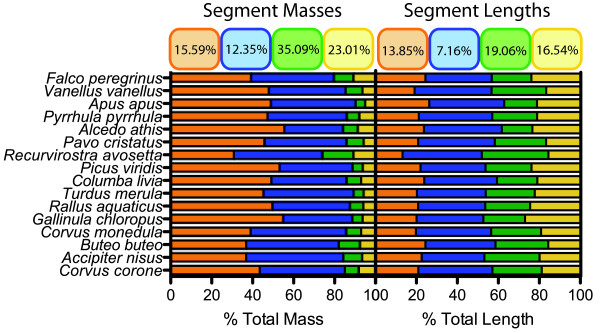
**Comparison of relative proportions of hindlimb segments in terms of mass and length for a subsample of 15 neognath birds.** Numerical values in colored boxes are the coefficients of variation for each segment. In bar plots, the data is stacked and non-overlapping so that sum values add to 100%. Data for segment lengths come from Stoessel et al. [15]. Note that digit length only refers to that of digit III. Colors of boxes and bars correspond to segments as labeled in Figure 3. Significant differences in coefficients of variation within one set of traits (mass or length) were determined using the methodology of Sokal & Braumann [114] (see text for Results).

Using a two-way ANOVA with log-transformed data [114], a comparison of coefficients of variation for segment masses finds that the variance significantly differs among the segments (*P* < 0.0001; Figure 7). Post-hoc pair-wise comparisons indicate that the proximal pair of segments significantly differs in coefficient of variation from the distal pair of segments (*P* < 0.0001). This is in stark contrast to how coefficients of variation differ among the segments with regards to their length. The tibiotarsus possesses the lowest coefficient of variation; however, the coefficients of variation do not significantly differ, though the test approaches significance (*P* = 0.0537). This particular result is likely due to the greatly reduced sample size compared to that of Stoessel et al. [15]. However, performing a pair-wise comparison nonetheless indicates that the coefficient of variation of the tibiotarsus significantly differs (*P* < 0.0001) from those of the other segments (which do not significantly differ from one another). It is worth noting though that exceptions may exist to the constrained limb proportions reported by Stoessel et al. [15], given that some fossil birds (e.g., Hesperornithiforms) have greatly elongate tibiotarsi relative to the femur and tarsometatarsus [6].

The differences in limb segment variation with regards to mass vs. length suggest that between segment variation in mass and length are decoupled. It thus appears that while activation-inhibition dynamics likely restrict the relative proportions of segment lengths in birds and other amniotes [112], such mechanisms do not influence the abundance or perhaps the density of the different tissues comprising the limb segments. This suggests that while developmental mechanisms influence segment lengths relative to one another, other aspects of limb design, such as muscle architecture and bone robustness, may be under greater influence from functional demands and specializations. Alternatively, between segment variation in segmental traits apart from lengths may be under the influence of differing developmental mechanisms or a combination of developmental and functional constraints.

## Conclusions

Scale effects within hindlimb segment masses of neognath birds are either positively allometric (when scaled against body mass) or isometric (when scaled against hindlimb length). These scale effects are paralleled within the subclade Land Birds, apart from shank, pedal, and tarsometatarsal segment masses scaling with positive allometry relative to hindlimb length. These results for Neognathae are at odds with previously reported scaling relationships between segment lengths and body mass, in which femur length and digit III length scale with isometry and tibiotarsal and tarsometatarsal length scale with positive allometry. Rather, the scaling of segment mass relative to body mass appears to have stronger ties to the scaling of long bone mass relative to body mass, especially in the case of more distal limb segments. The scaling of hindlimb segment masses likely explains the scaling of stride frequency with body mass and how large-bodied birds increase speed, whereas the negative allometry of hindlimb segment masses may be precluded by the mechanical demands placed upon the limb by locomotor and ecological function. Modeling trait evolution by branch length scaling reveals the influence of phylogeny on segment mass values; however, inherent uncertainty in the fitting of evolutionary models curtails any robust inferences of trait evolution. In spite of recent work indicating that developmental patterning through activation-inhibition dynamics governs limb linear proportions, variation in relative segment masses does not appear to be under the influence of activation-inhibition dynamics.

## Competing interests

The author declares that he has no competing interests.
